# Targeting intracellular *p*-aminobenzoic acid production potentiates the anti-tubercular action of antifolates

**DOI:** 10.1038/srep38083

**Published:** 2016-12-01

**Authors:** Joshua M. Thiede, Shannon L. Kordus, Breanna J. Turman, Joseph A. Buonomo, Courtney C. Aldrich, Yusuke Minato, Anthony D. Baughn

**Affiliations:** 1 Department of Microbiology and Immunology, University of Minnesota Medical School, Minneapolis, MN, USA; 2 Department of Medicinal Chemistry, University of Minnesota, Minneapolis, MN, USA.

## Abstract

The ability to revitalize and re-purpose existing drugs offers a powerful approach for novel treatment options against *Mycobacterium tuberculosis* and other infectious agents. Antifolates are an underutilized drug class in tuberculosis (TB) therapy, capable of disrupting the biosynthesis of tetrahydrofolate, an essential cellular cofactor. Based on the observation that exogenously supplied *p*-aminobenzoic acid (PABA) can antagonize the action of antifolates that interact with dihydropteroate synthase (DHPS), such as sulfonamides and *p*-aminosalicylic acid (PAS), we hypothesized that bacterial PABA biosynthesis contributes to intrinsic antifolate resistance. Herein, we demonstrate that disruption of PABA biosynthesis potentiates the anti-tubercular action of DHPS inhibitors and PAS by up to 1000 fold. Disruption of PABA biosynthesis is also demonstrated to lead to loss of viability over time. Further, we demonstrate that this strategy restores the wild type level of PAS susceptibility in a previously characterized PAS resistant strain of *M*. *tuberculosis*. Finally, we demonstrate selective inhibition of PABA biosynthesis in *M*. *tuberculosis* using the small molecule MAC173979. This study reveals that the *M*. *tuberculosis* PABA biosynthetic pathway is responsible for intrinsic resistance to various antifolates and this pathway is a chemically vulnerable target whose disruption could potentiate the tuberculocidal activity of an underutilized class of antimicrobial agents.

Tuberculosis (TB) causes over 1.7 million deaths per year and an estimated two billion people latently infected with *Mycobacterium tuberculosis* provides a large reservoir for ongoing reactivation and transmission of disease^1^,^2^. Treatment options for TB are longer and more complex than treatments for other bacterial infections. These complex therapies have led to patient non-compliance and disrupted treatment, resulting in an increased incidence of multidrug resistant and extensively drug resistant *M*. *tuberculosis* infections^1^,^3^. The search for new therapeutic options for treatment of drug susceptible as well as drug resistant strains has fueled an effort to repurpose existing drugs for TB therapy^4^,^5^.

Sulfonamides are broad spectrum antimicrobials commonly used to treat many bacterial infections^6^,^7^. These compounds are structurally similar to *p*-aminobenzoic acid (PABA), an essential precursor for synthesis of tetrahydrofolate. This structural similarity enables competitive inhibition of dihydropteroate synthase (DHPS) by sulfonamides thereby leading to disruption of tetrahydrofolate biosynthesis (Fig. 1a)^9^. In addition, it has been shown that sulfonamides can serve as alternative substrates for DHPS leading to the depletion of dihydropterin pools with the synthesis of dead end products that might further impair tetrahydrofolate biosynthesis^9^,^10^. Sulfonamides were used in early experimental TB therapy, but were replaced by the more potent anti-tubercular agents streptomycin and *p-*aminosalicylic acid (PAS)^11^,^12^,^13^. Dapsone, another PABA analog and DHPS inhibitor, is a cornerstone in treatment of infections with the related species *Mycobacterium leprae*. Yet, due to moderate potency and the extent of adverse drug reactions, this drug is not used for *M*. *tuberculosis* infections. Despite the limited utility of sulfonamides and dapsone against *M*. *tuberculosis* infection, enzymatic studies have confirmed that these compounds are potent competitive inhibitors for purified recombinant *M*. *tuberculosis* DHPS^10^,^14^. Evidence for the potentiation of sulfonamides has been demonstrated in the related organism, *Mycobacterium smegmatis*^15^. Thus, it is possible that more potent activity of antifolates can be revealed in *M*. *tuberculosis* through overcoming intrinsic resistance mechanisms.

DHPS inhibitors and PAS directly compete with PABA for binding to DHPS and consequently supplementation with PABA antagonizes the activity of these compounds in both whole cell and enzymatic assays^10^,^16^. Since PABA is an essential precursor for tetrahydrofolate biosynthesis, *M*. *tuberculosis* must maintain a basal level of intracellular PABA, as was confirmed by a recent metabolomic analysis of *M*. *tuberculosis*^17^. Importantly, these studies demonstrated a dose dependent increase in intracellular PABA in response to treatment with DHPS inhibitors and PAS^17^. However, the effect of intracellular PABA on the activity of these antimicrobial agents remains unexplored. We reasoned that since internal PABA levels are significantly increased following antifolate treatment, PABA synthesis might play a crucial role in intrinsic resistance to these drugs. Furthermore, we hypothesized that impairing synthesis of PABA would abolish this intrinsic drug resistance and lead to potentiation of antifolate action against *M*. *tuberculosis*.

## Results

### Disruption of PABA biosynthesis potentiates anti-tubercular antifolate action

**Figure 1:**
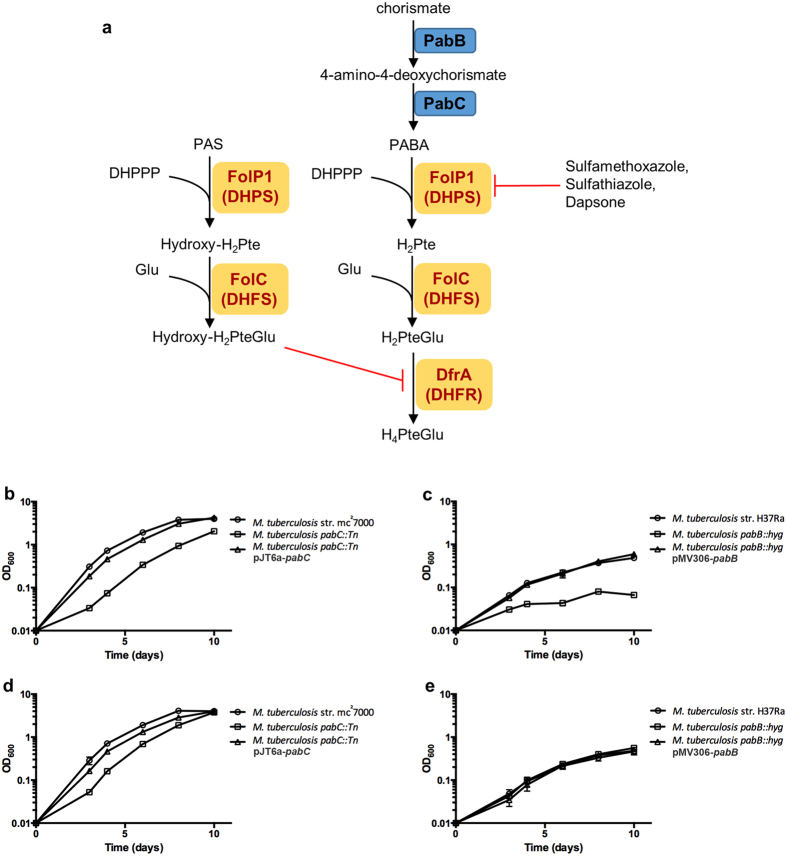
Disruption of PABA biosynthesis leads to growth defects in *M*. *tuberculosis*. (**a**) Schematic of tetrahydrofolate biosynthesis and PAS activation in *M*. *tuberculosis* and enzymatic targets of antifolate drugs as described by Minato *et al*.^8^. (**b**,**c**) The growth of indicated strains was assessed in PABA free 7H9 supplemented medium. Growth was determined by OD_600_ measurements taken every 2 days over a 10 day period. Growth curves were conducted in biological triplicate. Error bars denote standard deviation. (**d**,**e**) Exogenous PABA supplementation restores growth. The growth of indicated strains was assessed in PABA free 7H9 supplemented medium amended with 10 μg/ml PABA. Growth was determined by OD_600_ measurements taken every 2 days over a 10 day period. Growth curves were performed in biological triplicate. Error bars denote standard deviation.

To begin assessing the effects of intracellular PABA concentration on potency of DHPS inhibitors, *M*. *tuberculosis* transposon mutant strains deficient for PABA production were isolated. By screening a library of 5,000 independent transposon insertion mutants, we identified two strains that were unable to grow on PABA-free agar medium. These PABA auxotrophic strains were found to harbor transposon insertions in *pabC*, encoding the 4-amino-4-deoxychorismate lyase responsible for catalyzing the final step in PABA biosynthesis (Fig. 1a). Growth in PABA-free medium was assessed and a growth defect was observed in the *pabC*::Tn disruption strain relative to wild type (Fig. 1b). This growth defect was alleviated by expression of a wild type copy of *pabC* from an integrative mycobacterial vector (Fig. 1b) or with the addition of exogenous PABA to the growth medium (Fig. 1d). The minimum concentration of PABA necessary to restore wild type growth in the *pabC*::Tn strain was determined to be 5 pg/ml. While disruption of *pabC* caused auxotrophy on plates, the observed bradytrophic phenotype in liquid culture suggests a low level of sustained PABA production.

Next we examined the susceptibility of the *pabC*::Tn strain to the DHPS inhibitors sulfamethoxazole, sulfathiazole and dapsone (Table 1). The *pabC*::Tn strain showed a dramatic increase in susceptibility to DHPS inhibitors, ranging from 8 to greater than 500 fold, compared to susceptibility of the wild type strain. Importantly, these drugs also showed greatly enhanced bactericidal activity at biologically relevant concentrations against the *pabC*::Tn strain while remaining bacteriostatic against the wild type strain. Susceptibility to isoniazid was evaluated to determine whether enhanced drug susceptibility of this mutant strain was a general phenomenon or specific to anti-folate drugs. As anticipated, the isoniazid MIC_90_ was indistinguishable for the wild type and *pabC*::Tn strains, indicating that enhanced drug susceptibility associated with disruption of PABA biosynthesis was specific for anti-folates. These data indicate that inhibition of PABA biosynthesis can sensitize *M*. *tuberculosis* to otherwise less effective anti-folate drugs.

We sought to examine whether this potentiation effect could be applied to *p-*aminosalicylic acid (PAS), a second line drug commonly used to treat MDR TB^1^. PAS is also a structural analog of PABA and competes with PABA for activation by the concerted action of DHPS and dihydrofolate synthase. Once incorporated, PAS is able to poison folate metabolism likely through inhibition of dihydrofolate reductase (Fig. 1a)^17^,^18^. Similar to DHPS inhibitors, PAS anti-tubercular action can be antagonized by exogenously supplied PABA^13^,^16^. Thus, we reasoned that intracellular PABA levels could also provide intrinsic resistance to PAS. The PAS minimum inhibitory concentration (MIC) and minimum bactericidal concentration (MBC) for the *pabC*::Tn strain were both found to be reduced by greater than 1000-fold, relative to those for the wild type strain (Table 1).

### PabB is a novel, bactericidal target in *M*. *tuberculosis*

**Figure 2:**
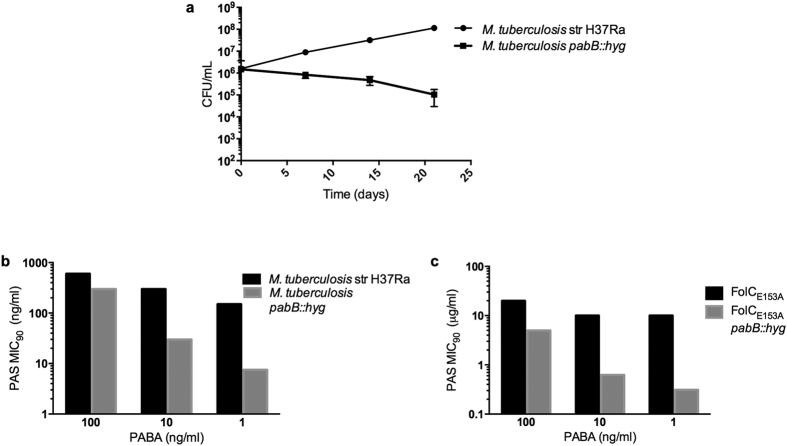
Disruption of PABA biosynthesis is bactericidal and restores susceptibility to PAS resistant strains. (**a**) Loss of *M*. *tuberculosis pabB::hyg* viability during PABA starvation. Strains were inoculated into PABA free 7H9 supplemented medium at an initial OD_600_ of 0.01 and serially diluted at 7 day intervals to determine remaining CFU. Data represent mean of biological triplicate samples with error bars denoting standard deviation. **(b,c)** PAS MIC_90_ decreases in PABA deficient drug susceptible strains **(b**) and drug resistant strains **(c)**. Indicated strains were inoculated into 7H9 supplemented medium amended with 1, 10, or 100 ng/ml of PABA to allow for growth. MIC_90_ was determined by OD_600_ for each strain at the indicated concentration of PABA and plotted along the Y-axis. Data shown are representative of biological triplicate samples for each strain at each PABA concentration.

The bradytrophic phenotype of the *M*. *tuberculosis pabC*::Tn strain suggests that PabC would not be an ideal stand-alone target, thus, we sought to determine whether disruption of alternative steps in PABA biosynthesis would result in a more severe defect. PABA production in *M*. *tuberculosis* occurs in a two-step pathway from chorismate. The *pabB* gene encodes a putative 4-amino-4-deoxychorismate synthase that catalyzes the amination of chorismate to afford 4-amino-4-deoxychorismate (ADC) while PabC catalyzes the subsequent conversion of ADC to PABA (Fig. 1a). Because the conversion of ADC to PABA could occur spontaneously through a concerted pericyclic pathway^19^, but the transformation of chorismate to ADC cannot occur spontaneously, we hypothesized that disruption of PabB would generate a stronger auxotrophic phenotype. We used the specialized transduction approach^20^ to construct a *M*. *tuberculosis pabB*::*hyg* strain by allelic exchange. The *pabB*::*hyg* strain was found to be auxotrophic for PABA on both solid agar and in broth culture (Fig. 1c). Unabated growth of this strain was restored by expression of *pabB* from a mycobacterial expression vector (Fig. 1c) as well as by supplementation of the growth medium with exogenous PABA (Fig. 1e). The minimum concentration of PABA required to restore wild type growth kinetics was determined to be 1 ng/ml. To determine whether the PABA auxotrophy resulted in bacteriostatic or bactericidal effects, we evaluated survival of the *pabB*::*hyg* strain in PABA-free broth culture. Culture viability, as assessed by determining colony forming units (CFU) per ml, decreased over time in PABA-free medium (Fig. 2a). These data suggest PABA synthesis as a potential target for discovery of bactericidal anti-tubercular agents. We then tested susceptibility of the *pabB*::*hyg* strain to PAS treatment. Since the *pabB*::*hyg* strain is auxotrophic for growth in broth culture, we performed PAS MICs in medium containing various concentrations of PABA. We found the PAS MIC to be dependent on the concentration of exogenous PABA (Fig. 2b). Similar to the *pabC*::Tn strain, deletion of *pabB* greatly potentiated PAS anti-tubercular action in limiting PABA conditions. Again isoniazid was included as a control. In line with the observations for the *pabC*::Tn strain, the level of isoniazid susceptibility between the wild type and *pabB*::hyg did not change (Fig. S1), supporting the idea that disruption of PABA biosynthesis specifically enhances anti-folate susceptibility in *M*. *tuberculosis*. Collectively, these data indicate that intracellular PABA levels play a role in intrinsic resistance to PAS, confirm PabB as a bactericidal target, and demonstrate synergy of PAS with deletion of *pabB*.

### Disruption of PABA synthesis restores PAS susceptibility in a resistant strain

As disruption of PABA synthesis drastically improves PAS efficacy, we reasoned that this strategy could restore susceptibility to PAS in a resistant mutant strain. Mutations in *folC*, encoding dihydrofolate synthase, confer PAS resistance in both laboratory and clinical isolates^18^,^21^,^22^. We deleted *pabB* in a previously described PAS resistant *folC* mutant containing a glutamate to alanine substitution at position 153 (*folC*_E153A_)^22^. The PAS MIC was determined using various concentrations of exogenous PABA. The *M*. *tuberculosis folC*_E153A_ strain was resistant to PAS at concentrations up to 20 μg/ml (Fig. 2c). However, disruption of *pabB* lowered the MIC to <0.156 μg/ml, a reduction greater than 2-fold below the PAS susceptible parental strain MIC during PABA limitation (Fig. 2c). These results demonstrate that targeting intrinsic resistance pathways can restore PAS susceptibility in resistant strains.

### Chemical targeting of PABA biosynthesis

**Figure 3:**
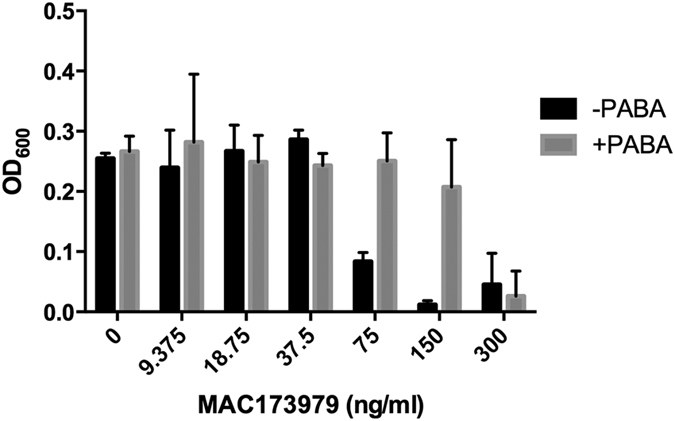
PABA biosynthesis is a chemically tractable target in *M*. *tuberculosis*. The ability of MAC173979 to inhibit growth of *M*. *tuberculosis* was determined using a 96-well plate format. *M*. *tuberculosis* mc^2^7000 was cultured to exponential phase, diluted to an OD_600_ of 0.01 and inoculated into 200 μl of supplemented M9 minimal medium with or without 10 μg/ml PABA. MAC173979 was added using a 2-log dilution scheme. Data shown represent the mean and standard deviation of three biological replicates.

Since not all biosynthetic pathways can be targeted by small molecules, we sought a chemical inhibitor of PABA biosynthesis to recapitulate our genetic findings. To date, no specific chemical inhibitor for *M*. *tuberculosis* PABA biosynthesis has been described. However, a recent high-throughput screen identified a compound, MAC173979, capable of inhibiting *E*. *coli de novo* PABA biosynthesis and growth^23^. We synthesized MAC173979 and tested the antimicrobial activity against *M*. *tuberculosis*. We found that treatment of *M*. *tuberculosis* with MAC173979 phenocopied deletion of *pabB*, inhibited growth with an MIC of 75 ng/ml and could be antagonized by supplementation with exogenous PABA (Fig. 3). Checkerboard assays were performed with MAC173979 and PAS, and the fractional inhibition concentration index (FICI) was calculated to assess drug interaction between these compounds (Table 2). In these assays, the FICI ranged from 0.5 to 0.87, indicating a mild synergistic effect.

## Discussion

Repurposing existing drugs offers an opportunity to fill the gap in the drug discovery pipeline^24^. The ability to improve drug efficacy through co-targeting strategies broadens the therapeutic tools available to treat infectious diseases. One well-known example of this augmentation is in the combined use of β-lactam antibiotics with clavulanic acid in order to neutralize β-lactamases^25^. However, enzymes capable of inactivating drugs are only one factor contributing to intrinsic drug resistance in bacteria. Other factors including membrane penetration, efflux mechanisms, and endogenous native substrate competition contribute to intrinsic drug resistance^26^,^27^,^28^. The ability to improve susceptibility of *M*. *smegmatis* to antifolates has been demonstrated previously through disruption of folate reserve pools^15^. In this work, we built on the concept of targeting intrinsic resistance by targeting production of an intracellular competitor in order to improve antifolate action. Through depletion of intracellular PABA levels via disruption of either *pabB* or *pabC*, we observed drastic enhancement, spanning several orders of magnitude, of *M*. *tuberculosis* susceptibility to DHPS inhibitors as well as PAS. Further characterization of PABA biosynthesis disruption through survival assays demonstrated loss of cellular viability over time in the absence of PABA, indicating PABA biosynthesis as a novel bactericidal target in *M*. *tuberculosis*.

As antimicrobial resistance is a major concern in *M*. *tuberculosis* infections, we examined the impact that co-targeting intrinsic resistance mechanisms has on re-sensitizing bacteria to drug treatment. Mutations in *folC* are one of the most common mechanisms of resistance to PAS found in clinical isolates^22^. We observed that susceptibility to PAS could be restored to below wild type levels by genetically disrupting PABA biosynthesis in a *folC* mutant strain, indicating that it is possible to circumvent previously developed resistance.

While many essential pathways might look like ideal drug targets, not all enzymes are suitable candidates for small molecule inhibitors^24^,^29^,^30^. For proof of concept, we synthesized the small molecule inhibitor MAC173979 to evaluate the PABA biosynthetic pathway as a cellular target in *M*. *tuberculosis*. We tested MAC173979 efficacy in the presence and absence of PABA to ascertain whether it was specifically targeting PABA biosynthesis. In line with observations made using *E*. *coli*, MAC173979 inhibited *M*. *tuberculosis* growth *in vitro* at nanomolar concentrations and targets PABA biosynthesis as exogenous PABA antagonized its action between 4 and 8-fold. Further, MAC173979 demonstrated mild synergy in combination treatment with PAS. Based on the genetic data, a stronger interaction may be expected between these drugs, however; it is not yet clear if there are additional undefined cellular responses to MAC173979 that may impact the anti-tubercular effects of PAS. Difficulties with MAC173979 highlight a need to develop more potent PABA biosynthesis inhibitors for further pursuit of potential antifolate co-treatment strategies.

Taken together, these data suggest that co-targeting intracellular competitor production opens new avenues for therapeutic interventions that take advantage of well-established antimicrobials. Targeting intrinsic resistance not only improves antimicrobial action, but offers a route to circumvent and prevent emerging drug resistance. Here we demonstrate the potential to improve the antitubercular potency of multiple antifolates by several orders of magnitude in both drug susceptible and drug resistant *M*. *tuberculosis*. Furthermore, as DHPS inhibitors are used to treat a wide range of infections, co-targeting PABA biosynthesis could be a viable option for improving treatment of many other types of infections. Developing novel therapeutic approaches to treat *M*. *tuberculosis* infection is paramount to combat the increasing prevalence of drug resistance.

## Methods

### Bacterial strains and growth conditions

Middlebrook 7H9 broth supplemented with 0.2% glycerol, 10% oleic acid-albumin-dextrose-catalase (OADC), 0.05% tyloxapol and 50 µg/ml pantothenate, or 7H10 agar medium supplemented with 0.2% glycerol, 10% OADC and 50 µg/ml pantothenate were utilized for *M*. *tuberculosis* strain mc^2^7000. Middlebrook 7H9 broth supplemented with 0.2% glycerol, 10% OADC and 0.05% tyloxapol, or 7H10 agar medium supplemented with 0.2% glycerol and 10% OADC were utilized to cultivate *M*. *tuberculosis* strain H37Ra. Kanamycin and/or hygromycin were added at 50 μg/ml or 150 μg/ml, respectively, when appropriate. PABA-free medium was made using glassware baked at 180 °C for at least one hour to remove any residual PABA. All strains used in this study are described in Table S1.

### Screening for transposon insertion mutants

Transposon mutagenesis was performed on *M*. *tuberculosis* strain mc^2^7000 as previously described^31^. Briefly, transposon mutants were isolated on supplemented 7H10 agar medium containing 10 μg/ml PABA and 50 μg/ml kanamycin. Colonies were picked and patched to supplemented 7H10 agar medium without or with 10 μg/ml PABA (Sigma-Aldrich). Mutants unable to grow on the PABA-free medium were selected for further evaluation. Transposon insertion sites were identified as previously described^32^.

### Cloning methods

For complementation of the *M*. *tuberculosis pabC* and *pabB* mutant strains, respective genes were cloned in the integrative mycobacterial vectors pJT6a^33^ containing a hygromycin resistance cassette or pMV306^34^ containing a kanamycin resistance cassette (Table S2). Briefly, the *pabC* and *pabB* genes were amplified by PCR using primers pabC_F and pabC_R, and, pabB_F and pabB_R respectively (Table S3). The amplicons and vectors were cut with HindIII and EcoRI and ligated together to produce pJT6a-*pabC* and pMV306-*pabB* respectively (Table S2). The recombinant plasmids were propagated in *E*. *coli* DH5α and maintained with hygromycin or kanamycin selection.

### Construction of deletion strains

A previously described allelic exchange system was utilized to replace the *pabB* locus with a hygromycin resistance cassette in relevant parental strains^20^. Briefly, the allelic exchange substrate containing flanking regions (~1000 bp) homologous to the flanking regions of *pabB* was constructed using plasmid p0004S (Table S2) to generate p1005. Flanking regions were amplified by the primer pairs: pabB_Up_For, pabB_Up_Rev and pabB_Dwn_For, pabB_Dwn_Rv (Table S3). This plasmid was ligated into the PacI site of the specialized transducing phage phAE159 (Table S2) to generate ph1005. ph1005 was propagated in *M*. *smegmatis* mc^2^155 to produce high titer phage. Phage ph1005 was then used to deliver the *pabB* deletion substrate into *M*. *tuberculosis* strain H37Ra using specialized transduction. Transductants were plated on supplemented 7H10 agar plates containing 10 μg/ml PABA and hygromycin to select for recombinant strains. Genomic DNA was prepared and deletion of *pabB* was verified using PCR and sequencing of respective amplicons.

### Growth experiments

Growth was assessed by measuring optical density at 600 nm (OD_600_). Initially, all strains were passaged in PABA-free supplemented 7H9 broth to titrate out PABA. Exponentially growing cultures were washed twice in an equal volume of PABA-free, supplemented 7H9 broth and diluted to an OD_600_ of 0.01 in PABA-free supplemented 7H9 broth for incubation at 37 °C. Supplemental PABA was added as indicated.

### Determining *pabB* loss of viability

Strains were passaged in PABA-free supplemented 7H9 broth to titrate out PABA. Exponentially growing strains were then washed twice in an equal volume of PABA-free supplemented 7H9 broth and diluted to an OD_600_ of 0.01 in PABA-free, supplemented 7H9 broth for incubation at 37 °C. The samples were serially diluted and plated on supplemented 7H10 plates containing 10 μg/ml PABA to determine CFU every 7 days over a 21 day time-course.

### Determination of antifolate MICs and MBCs

The minimum inhibitory concentration (MIC_90_) was defined as the concentration of drug required to inhibit 90% of growth relative to a no drug control. Growth was assessed by optical density (OD_600_). Exponentially growing strains were washed twice in an equal volume of PABA-free supplemented 7H9 broth and diluted to an OD_600_ of 0.01 in PABA-free supplemented 7H9. The PABA-free exponentially growing cultures were diluted to an OD_600_ of 0.01. Exogenous PABA was added at 100 ng/ml, 10 ng/ml, or 1 ng/ml as necessary for *pabB*::*hyg* strains. Drugs were added using a log_2_ dilution scheme, cultures were incubated at 37 °C and the OD_600_ was measured at day 14 to determine the MIC_90_. Cultures were also serially diluted and plated for CFU on supplemented 7H10 containing 10 μg/ml PABA on day 0 and day 14 to determine the minimum bactericidal concentration (MBC_99_) required to kill 99% of the initial population.

### Evaluation of antitubercular activity of MAC173979

MAC173979 was synthesized as described previously^23^ with minor modifications as described in the Supporting Information. M9 minimal medium supplemented with 0.4% glucose, 0.2% glycerol, 0.05% tyloxapol, and 50 µg/ml pantothenate was used for all experiments involving MAC173979. Initial MIC determination and PABA antagonism assays were conducted using a 96-well plate format. Briefly, mc^2^7000 was grown to exponential phase, diluted to an OD_600_ of 0.01 and inoculated into 200 μl of medium. MAC173979 was added using a log_2_ dilution scheme and 10 μg/ml of PABA was added as appropriate to determine antagonism. Cultures were incubated at 37 °C and the OD_600_ was measured at day 14 to determine the MIC_90_.

### Evaluation of synergy between PAS and MAC173979

Synergy was evaluated by performing checkerboard assays. Briefly, M9 minimal medium supplemented with 0.4% glucose, 0.2% glycerol, 0.05% tyloxapol, and 50 µg/ml pantothenate was used to culture mc^2^7000 to exponential phase. Upon reaching exponential phase, mc^2^7000 was diluted to an OD_600_ of 0.01 and inoculated into 5 ml of supplemented M9 medium. Bottles were arrayed as 7 rows with 10 columns. PAS was added to each column in a log_2_ dilution scheme with column 1 containing 300 ng/ml and column 10 as the no drug control. MAC173979 was then added in a log_2_ dilution scheme to each row with row 1 containing 120 ng/ml and row 7 as the no drug control. Bottles were incubated at 37 °C and the OD_600_ was measured at day 10. FICI was calculated using the following formula: ([MIC drug B in presence of Drug A]/[MIC of drug B]) + ([MIC of drug A in the presence of drug B]/[MIC of drug A])^35^.

## Additional Information

**How to cite this article**: Thiede, J. M. *et al*. Targeting intracellular *p*-aminobenzoic acid production potentiates the anti-tubercular action of antifolates. *Sci. Rep.* **6**, 38083; doi: 10.1038/srep38083 (2016).

**Publisher's note:** Springer Nature remains neutral with regard to jurisdictional claims in published maps and institutional affiliations.

## Supplementary Material

Supplementary Information

## Figures and Tables

**Table 1:** Antifolate efficacy against wild type and PABA-deficient *M*. *tuberculosis*.

Drug	wt MIC_90_ (μg/ml)	*pabC*::Tn MIC_90_ (μg/ml)	Fold-Change	wt MBC_99_ (μg/ml)	*pabC*::Tn MBC_99_ (μg/ml)	Fold-Change
Sulfamethoxazole	8	≤1	≥8	>64	<2	>32
Dapsone	64	≤0.13	≥512	128	<0.25	>512
Sulfathiazole	4	≤0.06	≥64	>64	<0.25	>256
PAS	0.3	≤0.000234	≥1000	0.6	0.0006	1000
Isoniazid	0.04	0.04	1	ND	ND	ND

MIC_90_, minimum concentration of drug required to inhibit 90% of growth relative to the respective no drug control; MBC_99_, minimum concentration of drug required to reduce the number of colony forming units by 99% with 14 days of exposure, relative to input colony forming units; ND, not determined.

**Table 2:** The combinatorial impact of PAS and MAC173979 on *M*. *tuberculosis in vitro*.

Antibiotic Combination	FICI Range	Interaction	Replicates
PAS/MAC173979	0.5–0.87	Mild synergy/Additive	3

FICI, fractional inhibitory concentration was calculated as described in *Methods*.
