# Supplementary material for: Targeting intracellular p-aminobenzoic acid production potentiates the anti-tubercular action of antifolates
Source: Sci Rep. 2016 Dec 1;6:38083. doi: 10.1038/srep38083 (PMC5131483; doi:10.1038/srep38083)
Supplement: Supplementary Information [file srep38083-s1.pdf]

Title: Targeting intracellular *p*-aminobenzoic acid production potentiates the anti-tubercular action of antifolates

Authors:

Joshua M. Thiede[1,a], Shannon L. Kordus[1,a], Breanna J. Turman[1], Joseph A. Buonomo[2], Courtney C. Aldrich[2], Yusuke Minato[1*] and Anthony D. Baughn[1*]

Author affiliations:

[1]Department of Microbiology and Immunology, University of Minnesota Medical School, Minneapolis, MN, U.S.A.

[2]Department of Medicinal Chemistry, University of Minnesota, Minneapolis, MN, U.S.A.

[a]denotes equal author contribution

*denotes corresponding authors; A.D.B abaughn@umn.edu, Y.M. yminato@umn.edu

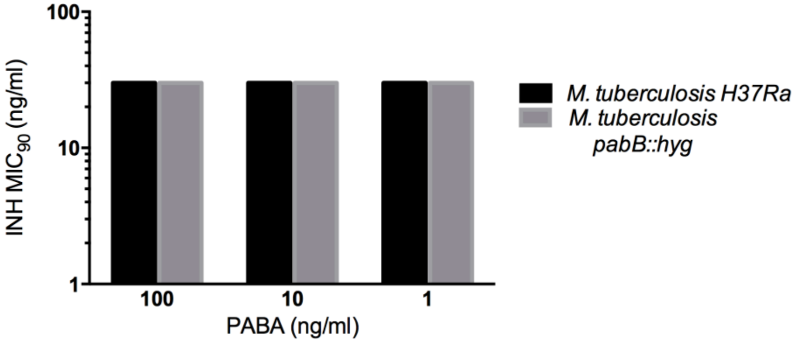

Figure S1. Disruption of PABA biosynthesis does not alter isoniazid susceptibility in *M. tuberculosis*. *M. tuberculosis* strains H37Ra and *pabB*::*hyg* were grown in supplemented 7H9 medium containing 1, 10, or 100 ng/ml of PABA. The minimum concentration of isoni[illegible]zid required to inhibit 90% of growth relative to the no drug control culture (MIC_{90}) was determined by measuring OD_{600} after 10 days of incubation. Data [illegible] biological [illegible]
.

Table S1. Bacterial strains used in this study.

Strain	Relevant Features	Source
M. tuberculosis mc^{2}7000	*M. tuberculosis* H_{37}Rv Δ*RD1* Δ*panCD*	1
M. tuberculosis pabC::Tn	*M. tuberculosis* mc^{2}7000 with a *magellan4* mini-transposon insertion in *pabC*	This work
M. tuberculosis pabC::Tn pJT6a::*pabC*	*M. tuberculosis* mc^{2}7000 *pabC*::Tn transformed with pJT6a-*pabC*	This work
M. tuberculosis H_{37}Ra	spontaneously attenuated derivative of *M. tuberculosis* H_{37}	2
M. tuberculosis pabB::*hyg*	*M. tuberculosis* H_{37}Ra with *pabB* coding sequence replaced by a hygromycin resistance cassette	This work
M. tuberculosis pabB::*hyg* pMV306-*pabB*	*M. tuberculosis* H_{37}Ra *pabB*::*hyg* transformed with pMV306-*pabB*	This work
M. tuberculosis folC$_{E153A}$	*M. tuberculosis* H_{37}Ra with PAS resistance mutation in *folC* (153Glu --> Ala)	3
M. tuberculosis folC$_{E153A}$ *pabB*::*hyg*	*M. tuberculosis* H_{37}Ra *folC*$_{E153A}$ with *pabB* coding sequence replaced by a hygromycin resistance cassette	This work
E. coli DH5α	*E. coli* strain used to propagate recombinant plasmids	4

Table S2. Plasmids used in this study.

**Name**	**Relevant Features**	**Source**
pJT6a-*pabC*	pJT6a containing the *M. tuberculosis* $H_{37}Rv$ *pabC* coding sequence under control of the P_{smyc1}tetO promoter/operator	This work
pMV306-*pabB*	pMV306 containing the *M. tuberculosis* $H_{37}Ra$ *pabB* promoter and coding sequence	This work
p0004S	Plasmid for construction of allelic exchange substrates	5
phAE159	Phasmid used to produce specialized transduction phage containing p0004S derived allelic exchange plasmids	6

Table S3. Oligonucleotide primers used in this study.

Name	Sequence	Source	Relevant Restriction Site
pabB_F	TTTTTT CCATGG ACG CCG AGC GTG CTT TTC CTA CT	This work	*Nco*I
pabB_R	TTTTTT AAGCTT CTA CCG CAC TTT GCT GGC TAA CC	This work	*Hin*dIII
pabC_F	TTTTTT AAGCTT ATG TTG AGG CAG ACG GGC GT	This work	*Hin*dIII
pabC_R	TTTTTT GAATTC TCA CCG GTC GCT GAC AAT AGC	This work	*Eco*RI
pabB_Up_For	TTTTTTTT CCATAAATTGG CTC GCA AAC TCG CGT CGT AGG	This work	*Pfl*mI
pabB_Up_Rev	TTTTTTTT CCATTTCTTGG GCA CCG GAC AGG CTC TCA TAC	This work	*Pfl*mI
pabB_Dwn_For	TTTTTTTT CCATAGATTGG AGT GTG GCA CCT GGT GTC CAC	This work	*Pfl*mI
pabB_Dwn_Rev	TTTTTTTT CCATCTTTTGG ACT CCA GCG CGT TAA CCG CAA	This work	*Pfl*mI

Supplemental Method. Synthesis of MAC173979:

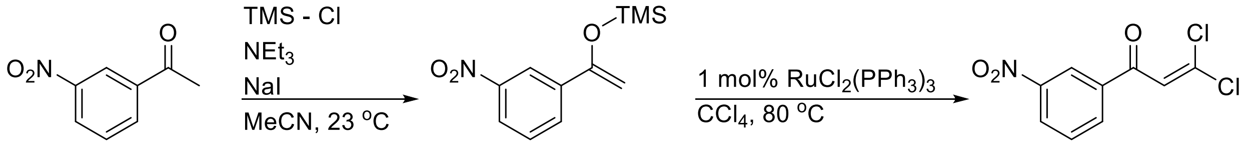

3,3-Dichloro-1-(3-nitrophenyl)prop-2-en-1-one (**MAC173979**) **Synthesis**. To a 25 mL round-bottom flask, equipped with a Teflon-coated magnetic stir bar, was added 3'-nitroacetophenone (991 mg, 6.0 mmol, 1.00 equiv.), trimethylsilyl chloride (0.951 mL, 7.5 mmol, 1.25 equiv.), and triethylamine (1.05 mL, 7.5 mmol, 1.25 equiv.). The mixture was stirred at 23 °C and a solution of sodium iodide (1.12 g, 7.5 mmol, 1.25 equiv.) in acetonitrile (7.5 mL, 1.0 M) was added dropwise, causing an exothermic reaction. After 30 min of stirring, the reaction was cooled to 0 °C, and cyclohexane (6 mL) was added. Then cold water (6 mL) was added and the layers were separated. The resultant aqueous layer was extracted with cyclohexane (2 × 6 mL). The combined organic layers were washed with cold H_2O (2 × 6 mL) and dried with anhydrous $MgSO_4$. The solvent was removed *in vacuo* to afford the intermediate trimethylsilyl enol ether as a yellow oil (1.21 g, 84%) that was used without further purification.

To a flame-dried 15 mL pressure tube, equipped with a Teflon-coated magnetic stir bar, was added CCl_4 (1.74 mL, 18.0 mmol, 3.60 equiv.) and the vessel was sealed with a silicon septum. The liquid was frozen at -78 °C and vacuum was applied to 0.3 mmHg. The solvent was then allowed to thaw under vacuum before the vessel was back-filled with argon. This freeze-pump-thaw process was repeated twice more. Ruthenium(II) tris-triphenylphosphine dichloride (57 mg, 0.05 mmol, 0.01 equiv.) and the trimethylsilyl enol ether prepared above (1.20 g, 5.00 mmol, 1.00 equiv.) were added under a blanket of argon. The vessel was sealed with a back-sealing screw cap, and then heated at 80 °C. After 17 h, the reaction was cooled to 23 °C, loaded directly onto a silica gel column (250 mL) and purified by flash chromatography (200 mL hexanes, followed by 80:20 hexanes–ethyl acetate). The title compound (857 mg, 70%) was collected as the first UV-active compound (R_f = 0.32, 80:20 hexanes–ethyl acetate) and separated from 3′-nitro-2,2,2-trichloroacetophenone (R_f = 0.30, 80:20 hexanes–ethyl acetate). The ^{1}H and ^{13}C NMR spectra matched previously published spectra.[7]

Supplemental References

1. Ojha, A. K. *et al.* Growth of *Mycobacterium tuberculosis* biofilms containing free mycolic acids and harbouring drug-tolerant bacteria. *Mol. Microbiol.* **69,** 164–174 (2008).

2. Steenken, W. Lysis of tubercle bacilli *in vitro*. *Exp. Biol. Med.* **33,** 253–255 (1935).

3. Zhao, F. *et al.* Binding pocket alterations in dihydrofolate synthase confer resistance to para-aminosalicylic acid in clinical isolates of *Mycobacterium tuberculosis*. *Antimicrob. Agents Chemother.* **58,** 1479–87 (2014).

4. Taylor, R. G., Walker, D. C. & McInnes, R. R. *E. coli* host strains significantly affect the quality of small scale plasmid DNA preparations used for sequencing. *Nucleic Acids Res.* **21,** 1677–1678 (1993).

5. Baughn, A. D. *et al.* Mutually exclusive genotypes for pyrazinamide and 5-chloropyrazinamide resistance reveal a potential resistance-proofing strategy. *Antimicrob. Agents Chemother.* **54,** 5323–5328 (2010).

6. Bardarov, S. *et al.* Specialized transduction: an efficient method for generating marked and unmarked targeted gene disruptions in *Mycobacterium tuberculosis*, *M. bovis* BCG and *M. smegmatis*. *Microbiology* **148,** 3007–17 (2002).

7. Zlitni, S., Ferruccio, L. F. & Brown, E. D. Metabolic suppression identifies new antibacterial inhibitors under nutrient limitation. *Nat. Chem. Biol.* **9,** 796–804 (2013).
